# Pathological reporting of cystectomy lymph nodes: a retrospective analysis of experience in Paris

**DOI:** 10.1007/s00345-021-03630-8

**Published:** 2021-03-20

**Authors:** André Oszwald, Gabriel Wasinger, Laura Larnaudie, Justine Varinot, Philippe Sebe, Olivier Cussenot, Eva Compérat

**Affiliations:** 1 Department of Pathology, Medical University of Vienna, Vienna, Austria; 2 Department of Pathology, GRC n°5 Predictive Onco-Urology, AP-HP, Tenon Hospital, Sorbonne University, 75020 Paris, France; 3 CeRePP, 75020 Paris, France; 4 Department of Urology, La Croix Saint-Simon, Paris, France; 5 Department of Urology, GRC n°5 Predictive Onco-Urology, AP-HP, Tenon Hospital, Sorbonne University, 75020 Paris, France

**Keywords:** Lymph node, Metastasis, Urothelial carcinoma, Size of metastasis, Sum of metastasis

## Abstract

**Purpose:**

Pathological evaluation of pelvic lymph node (LN) dissection (PLND) is important for management of cystectomy patients. However, challenges such as unclear interobserver variability of LN counting remain. Here, we assess interobserver variability of LN measures and their clinical utility, with a focus on variant histology.

**Methods:**

We retrieved radical cystectomy cases with PLND between 2010 and 2016 and reevaluated pathological parameters; number of total and metastatic LN, LN density (LND), length of metastatic LN and metastases, extranodal extension (ENE).

**Results:**

We report 96 patients: median age of 71a, 34 cases pN+, 36 cases with any extent of variant histology, median follow-up 10 months. Perivesical LN were only rarely identified, but frequently metastatic (4/9). Variant histology (34 cases) frequently exhibited LN metastasis (53% of pN+ cases). Interobserver variance was poor for total LN (kappa = 0.167), excellent for positive LN (0.85) and pN staging (0.96), and mediocre for LND (0.53). ROC analysis suggests that both LND and the sum of LN metastasis length may predict outcome (AUC 0.83 and 0.75, respectively).

**Conclusion:**

Our study confirms the notion of LND as a prognostic measure, but cautions due to strong interobserver variance of LN counts. The sum length of LN metastases could be a measure that is independent of LN counts. We find that microscopically identified perivesical LN merit particular attention. In summary, our study highlights current challenges in pathological reporting of PLND, confirms previous observations and forms a basis for further studies.

## Introduction

Radical cystectomy (RC) with pelvic lymph node dissection (PLND) is the standard treatment of urothelial carcinoma (UC) in muscle-invasive bladder cancer ($\ge$ pT2) [1] and a viable option for Bacillus Calmette–Guérin (BCG) refractory non-muscle-invasive UC (pT1) or carcinoma in situ [2, 3]. Reporting tumor stage (pT), surgical margins (R0/1), and lymph node (LN) status (pN) is essential to guide clinical decisions [4]. Pathological societies have recommended reporting extranodal extension (ENE) and the greatest diameter of lymph node metastases [5]. Taking into account these and other details could result in more accurate staging and stratification.

An unresolved challenge is the significance of lymph node counts in regard to patient management, which has recently been questioned [6]. Another controversial measure is the lymph node density (LND, ratio of positive LN to total LN), claimed to predict prognosis and better stratify LN-positive patients [7]. However, widely varying LND percentages have been reported [6, 7]. A further issue is a frequently requested minimum number of LN for diagnosis. All these measures suffer from the lack of established standards in gross handling, counting, and reporting of PLND specimens [8–10].

The aim of this study was to investigate interobserver variance of LN counts and the potential clinical relevance of several related assessments in the pathological report of UC. We assessed interobserver variability in enumerating total and metastatic LN (pN+) and LND. We also investigated differences between conventional and variant histology of UC. We find that total LN count and LND show higher interrater variability than absolute positive LN counts. This cautions against the use of LND due to inconsistent reporting and argues for strict criteria of LN assessment.

## Materials and methods

We retrospectively identified patients who underwent RC with PLND due to muscle-invasive or BCG-refractory disease between 2010/01/01 and 2016/11/30 in Hôpital Tenon APHP-Sorbonne and Hôpital des Diaconesses and retrieved the cases from our archives. PLND was defined as either limited or standard based on the recommended nomenclature of the EAU [11]. Since we could not retrospectively determine the precise location of common iliac LN relative to the ureter, we classified all PLND with respective LN as “standard or extended”.

Grossing protocols adhered to published standards [12]. The entire PLND was included, otherwise all palpable LN were included entirely, or divided if larger than 1 cm and embedded entirely. Standard sections were prepared (3 µm; HES: hematein, eosin, saffron). Interobserver variability was evaluated by reassessment of all cases in blinded manner (by authors EC and LL) and comparison to the initial pathology report using Cohen’s kappa, %-agreement and Pearson correlation. Only LN fulfilling histological criteria were counted (capsule, sinus, contiguous lymphoid tissue). We assessed numbers of total and metastatic LN, diameter of the LN metastasis, presence of ENE.

We retrospectively collected clinicopathological parameters including age, sex, tumor pTNM status, grade and stage, UC histology, follow-up regarding survival, death of disease and recurrence.

Statistical analyses were performed using specific packages within R Studio [13–16]. Comparisons between groups were performed using a two-tailed Kruskal–Wallis test and a significance level defined at *p* = 0.05 unless specified.

## Results

### Summary of cases and PLND

We included 96 patients with RC and PLND (median age 71a, male-to-female ratio 3.8:1, Table 1). Eighty-three cases were muscle-invasive UC, 34 cases exhibited LN metastases (pN+); 13 displayed positive surgical margins. Median follow-up was 10.3 months (range 0–65), during which 23 patients died of disease, and 13 patients experienced recurrence but survived. Variant histology component of any extent was present in 36 cases (38%).

**Table 1:** Summary of clinical and histological patient data

Clinical and histological patient data	All	pN+	Variant
Number of patients	96	34	36
Age in years, median (min.–max.)	71 (41–88)	68 (53–84)	71 (52–85)
Sex ratio (M/F)	3.8 (76/20)	3.1 (25/8)	3 (27/9)
NOS	60	14	
Variant histology—urothelial	36	12	
Micropapillary	9	8	
Nested	3	2	
Poorly differentiated	3	1	
Sarcomatoid	2	1	
Plasmacytoid	1	0	
Lymphoepithelioma-like	1	0	
Variant histology—non-urothelial	16	8	
Squamous	12	5	
Glandular	3	2	
Neuroendocrine	1	1	
pT0	1	0	0
pTa	2	0	0
pT1	10	0	0
pT2	17	3	3
pT3	42	18	21
pT4	24	12	12
pN0	62		16
pN1	10		6
pN2	22		12
pN3	2		3
R0	83	27	29
R1	13	7	7
Follow-up, months, median (min.–max.)	10.3 (0–65)	10 (0–55)	7.8 (0–39)
DOD	23	10	6

**Fig. 1:**
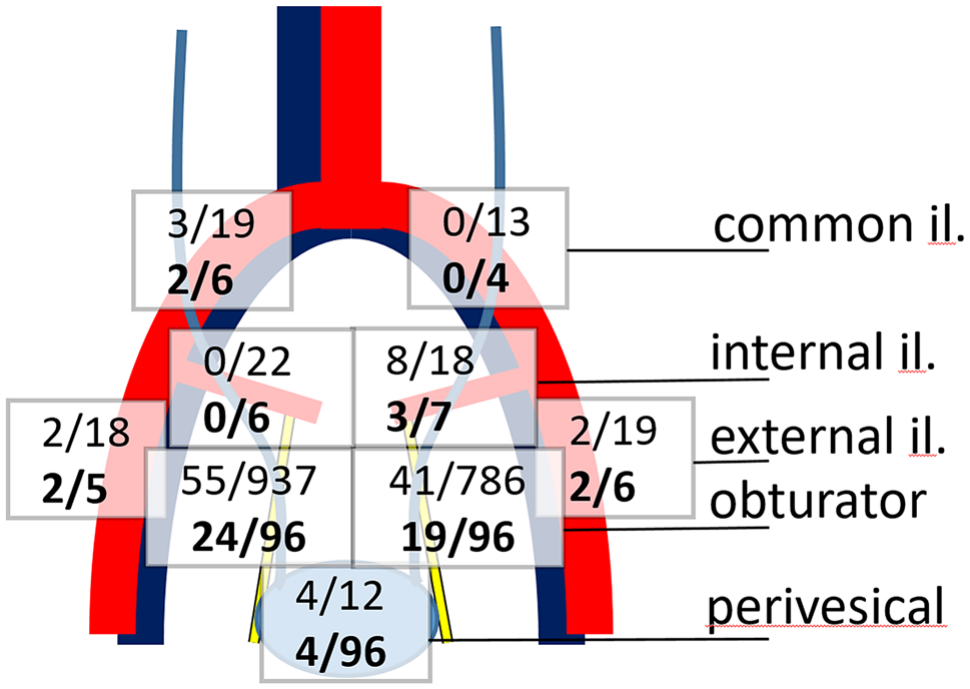
Distribution of LN by location. The top line indicates the absolute counts of positive LN/total LN counted over all cases. The bottom line (bold) indicates the number of patients with positive LN in this region/the number of patients where lymph nodes from this region were submitted

Seventy-nine patients underwent limited, 18 patients underwent standard or extended PLND (Fig. 1). Thirteen patients received neoadjuvant chemotherapy. We observed similar total LN counts between limited and non-limited templates (medians and ranges: 18 (1–54) vs 20 (8–39), *p* = 0.48). In contrast, positive LN counts were significantly higher in non-limited templates than in limited templates (medians and ranges: 0 (0–9) vs 2.5 (0–10), *p* = 0.02). No difference was found between patients who did or did not receive neoadjuvant chemotherapy regarding counts of total (*p* = 0.63) or positive (*p* = 0.12) lymph nodes.

The most frequent localizations of positive LN were obturator and internal iliac (30% of cohort). Perivesical LN were only found in nine cases, but displayed metastasis in four cases. Patients only rarely underwent PLND involving external (eight cases, three positive) and common iliac (ten cases, two positive) LN. Sixteen patients (17%) had positive lymph nodes in more than one region (station or side), five patients (5%) had positive disease in more than one lymph node station (i.e., obturator, perivesical, internal, external or common iliac).

### Variant histology and LN

**Fig. 2:**
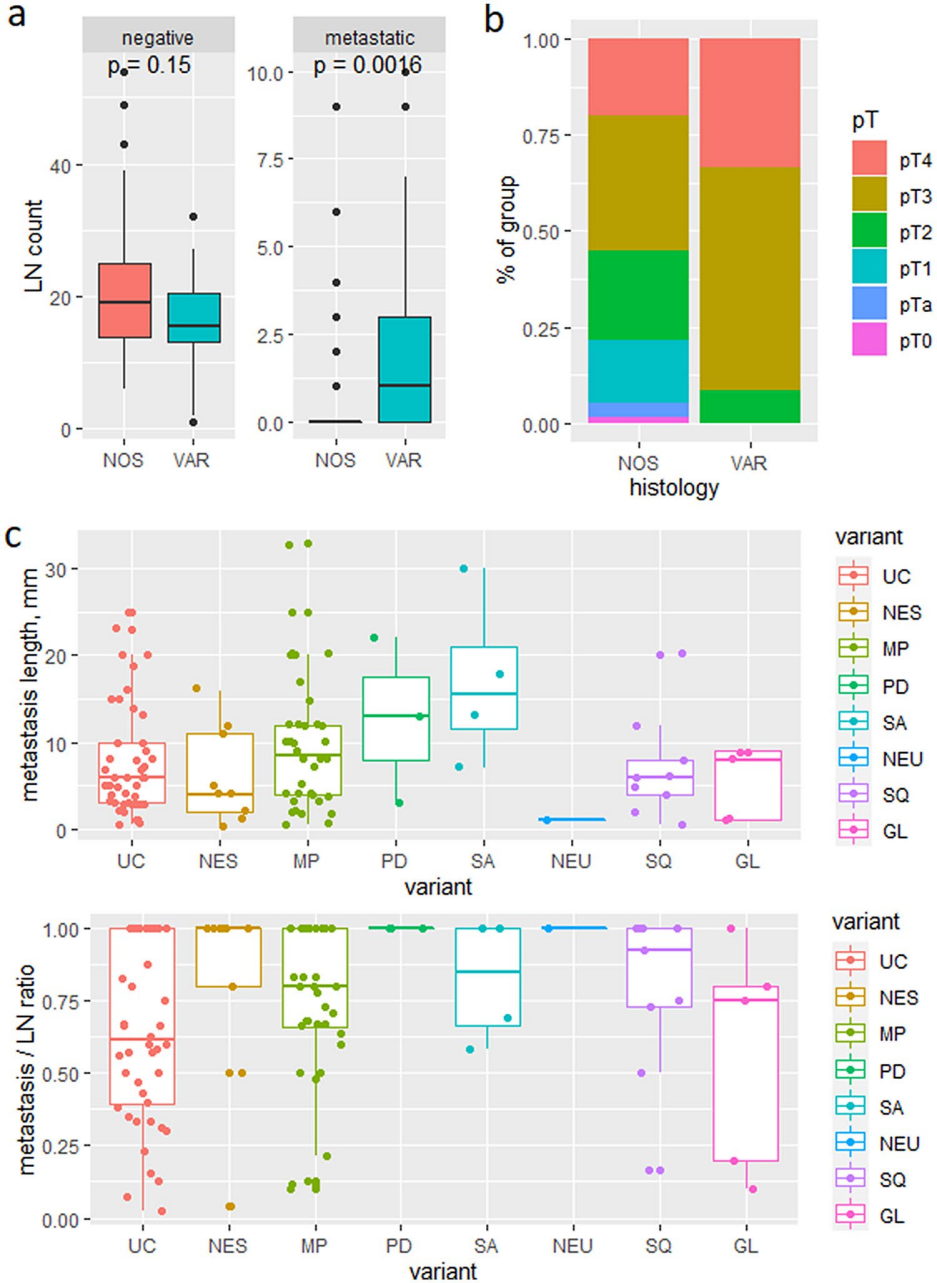
Comparisons between urothelial histology and variant histology. Metastatic LN counts are greater in the group showing variant histology component (**a**). These tumors also exhibit greater pT stage upon resection (**b**). Length of metastases and ratio of metastasis to LN length are shown (**c**)

Among pN+ cases, variant histology was overrepresented (59% in pN+ vs 38% in all cases). Vice versa, cases with variant histology were more frequently pN+ than not otherwise specified (NOS) cases (56% vs 23%, respectively) and had more positive LN (mean 1.9 vs 0.8, *p* = 0.0016) (Fig. 2a) and LND (0.13 vs 0.04, *p* = 0.0014). Concordantly, variant histology tumors presented with higher pT stage (*p* < 0.001) (Fig. 2b). Positive LN from patients with variant UC showed a higher ratio of LN metastasis to LN length (mean 0.77 vs 0.64, *p* = 0.019) and more frequent extranodal extension (one-sided Fisher’s exact test, *p* = 0.042). Among variant UC, two were frequently pN+ compared to the overall rate of 34%: micropapillary UC (9/10 pN+, all with ENE) and squamous cell carcinoma (6/14 pN+, 2 with ENE). We observed a trend of different length metrics in specific variant histology, but refrained from testing due to low case numbers (Fig. 2c).

### Interobserver variability

To evaluate interobserver variability, we calculated metrics comparing first and second LN assessment. We found a consistent mean difference in total LN count between observers, regarding the entire study cohort, or pN0 or pN+ patients (mean and SD of difference: 4.1 ± 4.4, 4.1 ± 4.2, 4.1 ± 4.8, respectively). The mean difference in positive LN count was 0.11 ± 0.41 (overall) and 0.32 ± 0.64 (pN+ cases). Importantly, the difference in positive LN count impacted tumor N staging in only two of 96 cases (pN0/pN1 and pN2/pN3).

Interobserver metrics were poor for total LN counts (20% agreement, kappa 0.167, *r* = 0.87). In contrast, metrics were excellent for positive LN counts (92% agreement, kappa 0.85, *r* = 0.99) and resulting tumor pN stage (98% agreement, kappa 0.96, *r* = 0.99). Mean lymph node density differed by 1.7 ± 5.1% (overall) and 4.7 ± 7.7% (pN+ cases). Of particular note, LND interrater metrics (agreement 73%, kappa 0.53, *r* = 0.96) were lower than for positive LN count or pN staging.

### Comparison of lymph node parameters

**Fig. 3:**
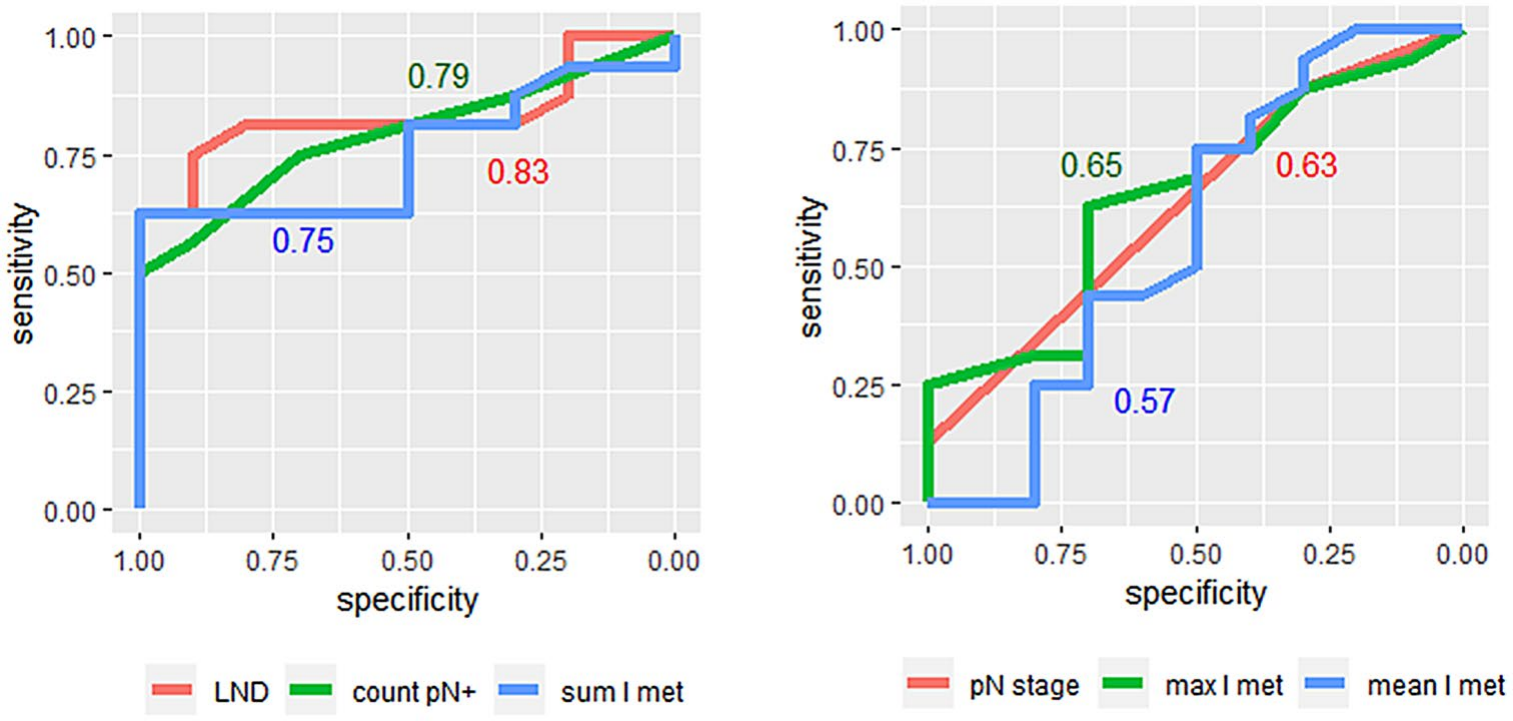
ROC analysis of LN assessments. LND, pN+ count and sum length of metastases show high AUC (stated in graph), pN stage, maximum or mean length of metastasis show lower AUC

To assess the common utility of various LN parameters, we performed ROC analysis regarding good or bad outcome (defined as cancer-related death or recurrence within the follow-up period). We found that LND, positive LN count and total length of LN metastases were good predictors of outcome (AUC 0.83, 0.79, and 0.75, respectively). We observed lower performance for the single largest or mean size of LN metastases and pN stage (Fig. 3).

## Discussion

LN metastasis is a predictor of poor outcome in bladder UC [17]. LN counts and the resulting LND likely suffer from the lack of established standards in gross handling, counting and reporting of PLND specimens [8–10]. The pathologist’s practical approach is to palpate and dissect LN from the surrounding adipose tissue. In inguinal LN, adipose or fibrotic transformation and tortuous configuration are frequent, requiring meticulous dissection [18]. Without a rigorous grossing protocol, accurate enumeration of LN is challenging due to the possibility of counting a LN multiple times. Microscopic counts usually differ from grossing; adipose LN are typically enlarged but frequently lack microscopic criteria of a LN (e.g., capsule, sinus) or encompass several small LN.

Our data show that interrater variability ranges from very poor (total LN) to excellent (total positive LN, pN staging) for different LN metrics. As a result, LND variability was intermediate. We suspect that the lack of rigorous histological criteria for counting LN are responsible for the poor performance of total LN counts, in addition to factors reported previously [10, 18].

Next, our data highlight the increased metastatic potential among specific types or variants of UC [19]; this report therefore supports the recommendation of reporting histological variants and tumor types regardless of their extent. In particular, micropapillary carcinoma (representing 10% of cases in our study) seems to exhibit highly aggressive behavior. The importance of extranodal extension (ENE) is under debate [20, 21]; we observed ENE more frequently in pN+ from variant histology cases. In our cohort, variant histology was more frequent than in previous reports, underlining that the actual prevalence is uncertain—possibly because reporting of variants differs between pathologists and departments [19].

A few studies have addressed the most frequent localizations of LN metastases in UC. In our study, the most frequent localization of pN+ were the ilio-obturator LN, with no preference for either side. We found that perivesical LN can only rarely be identified; however, if identified, they are frequently positive. Therefore, pathological identification of a perivesical LN may merit additional section levels due to their higher chance of being positive.

We found that the lymph node density (LND) has the potential for good prediction of outcome (AUC = 0.83). Despite this, we confirm that due to interrater variability, LND should be interpreted with caution, in accordance with a previous report [22]. This could be improved by adherence of pathologists to strict histological criteria when counting LN. We find that the total positive LN count is also a good predictor, and less affected by interobserver variability. Some organizations have recommended reporting the size of the largest metastasis [5], but only little has been reported on the total metastatic length [23]. This measure could be independent of subjective enumeration, and needs to be evaluated in future studies. Of note, LN metastasis length has been incorporated into pTNM staging of breast cancer [24].

The current EAU guidelines [25] state that every RC should be accompanied by an LND, but that there is little evidence of a survival benefit of extended or super-extended over standard or limited templates. The rationale for a standard template is that almost all pN+ cases will be identified, while the greater templates are likely to remove more positive LNs which would otherwise be left in place [26]. This rationale is supported by our finding that non-limited templates will likely yield a greater count of positive LN. On the other hand, there exists the possibility that in our cohort, non-limited templates were preferentially performed in patients with suspected pN+ disease. Considering the high interrater variability of LN counts, an extended LN template may be more important for accurate staging of pN+ cases than the actual pathological enumeration of LNs.

In our study, we have considered several factors both routinely and only rarely included in pathological reporting. Based on our results, we highlight the need for more unified criteria in LN counting, which are a problem for otherwise potentially useful measures such as LND. Furthermore, we show that absolute LN metastasis length may be a comparable but more objective measure of LN metastasis, awaiting closer investigation in future studies. We also highlight that rare perivesical LN identified via microscopy have a relatively high chance of bearing metastasis. Lastly, we underline the increased metastatic potential of variant histology bladder cancer and the recommendation of mentioning any component in the pathology report.

## Limitations

Limitations stem from our retrospective study design and the small cohort with strongly unequal portions of limited, standard and extended PLND. Our follow-up period is too short to make conclusions regarding prognostic thresholds, and we refer to outcome only to compare between LN parameters. The portion of cases with variant histology is higher than in previous reports; however, we find no reason for potential selection bias.
